# Theory of Connectivity: Nature and Nurture of Cell Assemblies and Cognitive Computation

**DOI:** 10.3389/fncir.2016.00034

**Published:** 2016-04-29

**Authors:** Meng Li, Jun Liu, Joe Z. Tsien

**Affiliations:** ^1^Brain and Behavior Discovery Institute and Department of Neurology, Medical College of Georgia at Augusta UniversityAugusta, GA, USA; ^2^The Brain Decoding Center, Banna Biomedical Research Institute, Yunnan Academy of Science and TechnologyYunnan, China

**Keywords:** nature vs. nurture, theory of connectivity, cell assembly, memory engram, generalization, imagination, motor control, decision-making

## Abstract

Richard Semon and Donald Hebb are among the firsts to put forth the notion of *cell assembly*—a group of coherently or sequentially-activated neurons—to represent percept, memory, or concept. Despite the rekindled interest in this century-old idea, the concept of cell assembly still remains ill-defined and its operational principle is poorly understood. What is the size of a cell assembly? How should a cell assembly be organized? What is the computational logic underlying Hebbian cell assemblies? How might Nature vs. Nurture interact at the level of a cell assembly? In contrast to the widely assumed randomness within the mature but naïve cell assembly, the *Theory of Connectivity* postulates that the brain consists of the developmentally pre-programmed cell assemblies known as the functional connectivity motif (FCM). Principal cells within such FCM is organized by the power-of-two-based mathematical principle that guides the construction of *specific-to-general combinatorial* connectivity patterns in neuronal circuits, giving rise to a full range of specific features, various relational patterns, and generalized knowledge. This pre-configured canonical computation is predicted to be evolutionarily conserved across many circuits, ranging from these encoding memory engrams and imagination to decision-making and motor control. Although the power-of-two-based wiring and computational logic places a mathematical boundary on an individual’s cognitive capacity, the fullest intellectual potential can be brought about by optimized nature and nurture. This theory may also open up a new avenue to examining how genetic mutations and various drugs might impair or improve the computational logic of brain circuits.

Semon (1904) and Hebb (1949) are among the firsts to explore the concept of *cell assembly*: a group of neurons that fire transiently or sequentially, as the computational primitives to encode an object, concept, or memory engram (Figure 1A). This idea is now best summarized as “fire together, wire together” (Löwel and Singer, 1992). But in the real brain, neurons fire spontaneously, typically with huge variations even during resting periods. Such spontaneous firing variability make it nearly impossible to determine real-time sequential firing patterns while billions of neurons in the brain are not silent (Legendy, 1967; Palm, 1987; Harris et al., 2003; Harris, 2005; Dragoi and Buzsáki, 2006; Pastalkova et al., 2008; Takehara-Nishiuchi and McNaughton, 2008; Buzsáki, 2010).

**Figure 1:**
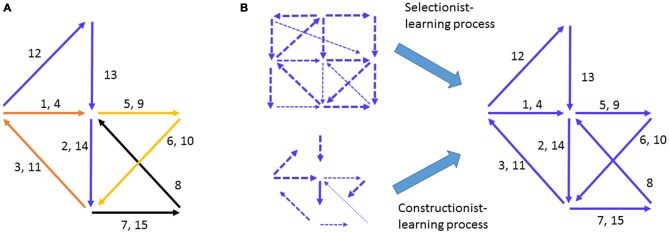
**Hebbian cell-assembly and the proposed mechanisms of its formation. (A)** Hebb illustrated his idea on the firing of the cell assembly as a way to represent concept or percept as follows: “*Any frequently repeated, particular stimulation will lead to the slow development of a “cell-assembly,” a diffuse structure comprising cells in the cortex and diencephalon, capable of acting briefly as a closed system, delivering facilitation to other such systems and usually having a specific motor facilitation. …The theory is evidently a form of connectionism…*” Different numbers represent the different neural pathways. Arrows represent a simple “assembly” of neural pathways and their firing chains or information flow. The drawing is adopted from Hebb (1949). **(B)** The mechanisms proposed to explain how Hebbian cell assembly may form. The *Selectionist Theory of Learning* vs. *Constructionist Theory of Learning* offered the two major ideas for the growth and maturation of cell assemblies, despite the fact that the internal organization of representational cell assembly was not defined.

## Is Connectivity of Cell Assembly Randomized Or Organized?

Two influential theories have taken center stage in explaining how the brain may develop assembly-level mental representations of external worlds. One is termed the *Selectionist Theory of Learning* (Changeux and Danchin, 1976; Edelman, 1993). The idea is that during development, the genetic program initiates multiple waves of neural growth and synapse overproduction and is then subjected to regressive selection, or *Neural Darwinism*, during learning via synaptic pruning and stabilization (Figure 1B). The other complementary theory is the *Constructivist Theory of Learning*, which postulates that learning interplays with the growth of neural connections over the prolonged postnatal developmental period to gradually construct representational networks (Figure 1B; Quartz and Sejnowski, 1997). By either assuming random patterns or overlooking what the innate patterns should look like as Hebb had originally done, both theories focused on the requirement of learning to construct yet undefined “*representational patterns*”. However, models based on random connectivity face the difficulty of explaining the natural emergence of innate cognitive abilities in infants in the absence of apparent learning (Carruthers et al., 2005).

## Theory of Connectivity: Canonical Computation of Cell Assemblies

How should cell assembly organize itself so that incoming information can be orderly and gradually converted into memory, concepts, and flexible motor behavior? We previously uncovered that CA1 cells used specific-to-general combinatorial strategy to encode three distinct fearful experiences (Lin et al., 2005, 2006; Tsien, 2007; Tsien et al., 2013). This seed of an idea led to the *Theory of Connectivity* that the specific-to-general combinatorial strategy may reflect the mathematical principle underlying the general organization of cell assemblies in the brain (Tsien, 2015). This theory described a “*power-of-two*” based, *specific-to-general* wiring logic, and predicts a series of pre-configured, conserved functional connectivity motifs (FCMs) capable of discovering specific features, as well as all possible relational patterns and abstract knowledge (Tsien, 2015, 2016).

**Figure 2:**
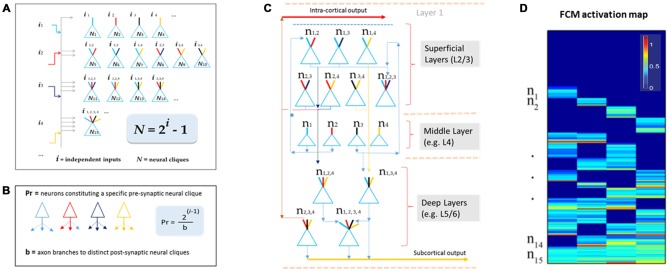
**The *Theory of Connectivity* predicts the existence of specific-to-general, combinatorial wiring logic across the brain. (A)** The proposed functional connectivity motif (FCM) is illustrated in a non-recurrent, feed-forward circuit. By following the proposed equation of *N* = 2^*i*^−1, the FCM exemplified here consists of 15 distinct neural cliques (*N*_1...15_), which cover all possible connectivity patterns in order to process four distinct inputs (*i_1_*, *i_2_*, *i_3_*, *i_4_*). The exponent *i represents* the number of distinct information inputs, and *N* is the number of neural cliques with all possible combinatorial connectivity patterns. **(B)** The number of presynaptic neurons (Pr) required to cover postsynaptic convergence can also be mathematically assessed (see the equation in the highlighted blue block, *Pr* = [2^i−1^]/b). For example, assuming a presynaptic neuron from the upstream FCM has only a single axon (branch number, *b* = 1) contacting a single postsynaptic neurons located in the downstream FCM containing 15 cliques (based on *i* = 4), the total number of such presynaptic specific-feature cells (providing input *i*_1_) required for comprehensively covering the specific-to-general, combinatorial-convergent postsynaptic cells in a non-recurrent downstream FCM would be eight. These eight postsynaptic neurons correspond to *N*_1_, *N*_5_, *N*_6_, *N*_7_, *N*_11_, *N*_12_, *N*_14_, *N*_15_ listed in the Panel **A**. On the other hand, if a single pre-synaptic neuron can have eight branches (*b* = 8; each making a contact with a unique postsynaptic neuron, respectively), this neuron is sufficient to provide inputs onto *N*_1_, *N*_5_, *N*_6_, *N*_7_, *N*_11_, *N*_12_, *N*_14_, *N*_15_ listed in Panel **A**. **(C)** The FCM-based computational logic can be mapped onto the multi-layered cortex as the general-purpose algorithm underlying canonical cortical computation. In the classic six-layered (L) cortex, L1 contains scattered interneurons and mainly dendrites and afferent axons from lower layers. L2 to L6 are the primary sites for canonical computation. The specific cliques occupy layer 4 (L4), which projects to L2/3 to form the initial set of combinatorial connectivity patterns (e.g., mostly two-featured combinations and some three-featured combinations). These subgeneral cliques then project downward to the deeper layers, L5/6, for generating even greater combinatorial connectivity. **(D)** Schematic “bar-code” illustrates the specific-to-general activation responses from the 15 distinct neural cliques (*n*_1−15_), processing four distinct inputs (*i* = 4). The warm color represents its activation level (% maximal activation). The cartoon illustration was adapted from Tsien (2015), *TINS*.

This theory defines the size of a cell assembly: each FCM consists of neural cliques (*N*) made of principal neurons receiving specific afferent inputs, as well as those principal neural cliques receiving progressively more convergent inputs that are comprehensively and combinatorially arranged, following the formula of *N* = 2^*i*^−1 (*i* for numbers of distinct information inputs, *N* is the number of neural cliques with all possible combinatorial connectivity patterns; Figure 2A).

As the evolutionarily conserved cell-assembly wiring logic, it should exhibit: (1) Anatomical prevalence—to be prevalent across neural circuits, regardless of gross anatomical shapes; (2) Species conservancy—to be conserved across different animal species; and (3) Cognitive universality—to be used as a universal computational logic for processing various cognitive information, including appetitive, social and/or fearful events. It should be noted that in the natural world situation, as *i* become increasingly larger in more intelligent animal species, *N* in a given cell-assembly can approximate to 2^i^ (e.g., ~90 or 95%) rather than rigidly calculated by the equation, this mathematical approximation would still preserve the essence of specific-to-general combinatorial logic.

## FCM Implemented in the Non-Recurrent, Feed-Forward Circuits

The proposed connectivity logic can be used to examine biological and mathematical boundaries of wiring efficiency or computational capacities using modeling (McClelland and Rogers, 2003; Cutsuridis et al., 2010; Stevens, 2012), including recurrent and non-recurrent networks. In the non-recurrent feed-forward network (e.g., the CA1 region or dentate gyrus [DG]), specific-to-general combinatorial patterns can be established by combinatorial wiring of the presynaptic upstream inputs (Figure 2A). This wiring logic produces instantaneous pattern-separation and pattern-generalization by a cell assembly, which is radically different from pattern separation or pattern completion that was specifically or sequentially assigned to one of the hippocampal subregions (Marr, 1971; Yassa and Stark, 2011; Rolls, 2013). Furthermore, in the CA3 which is viewed as a completely auto-associative recurrent network, the traditional theories would predict the loss of the specific-to-general neural-clique connectivity logic, because any specific input into specific cliques would easily be taken over by feedback inputs from its subgeneral and general cliques.

One of the critical factors in building computational models based on this wiring logic is to determine how specific input-neurons (presynaptic neurons, whether pyramidal cells and long-range inhibitory projection cells) should project onto the non-recurrent networks. For instance, the number of presynaptic specific input-neurons (Pr) needed to cover all distinct postsynaptic neural cliques can be estimated by using the equation of *Pr* = [2^i−1^]/b (Figure 2B), whereas *b* represents numbers of axon branches. By assuming that the presynaptic neuron has one axon branch which projects to one postsynaptic cell, one would expect that the minimal number of cells from the pre-synaptic specific neural clique should be eight (*Pr* = 8) to ensure the specific-to-general combinatorial coverage in the postsynaptic FCM that deals with four distinct information input (*i* = 4). Obviously, most neurons have more than one axon and each can bifurcate at multiple points along its length to multiple terminal locations to form synapses with several postsynaptic target cells. For example, a single pre-synaptic cell can be modeled to have eight axon branches, each projecting to the eight distinct post-synaptic clique cells (Figure 2B, only three branches are drawn). In the real brain, the actual number of neurons per clique and their axon branching patterns would depend on molecular genetics and local cues during neural development. The connectome projects can prove to be highly valuable for this type of analysis.

## Using Cortical Layers to Execute FCM-Based Canonical Computation

The cortex has a remarkably uniformed multi-layered architecture (usually with three or six layers; Barbas, 2015; Fournier et al., 2015). It has been shown that the cortex varies its surface area by a factor of 10,000 across a large number of surveyed mammalian species, while the thickness of cortical layers varies only by a factor of 10 (DeFelipe et al., 2002; Rakic, 2008). This finding has contributed to the search for a fundamental cortical “processing unit” (Hubel and Wiesel, 1977; Mountcastle, 2003). In literature, a variety of canonical neural computations have been proposed, including exponentiation, linear filtering, normalization, receptive field selectivity, gain control, etc. (Reichardt et al., 1983; Heeger, 1992; Bizzi et al., 1995; Hanes and Schall, 1996; Pouget and Snyder, 2000; Smith and Ratcliff, 2004; Carandini et al., 2005; Cisek, 2006; Kouh and Poggio, 2008; Carandini and Heeger, 2011; Miller, 2016). Despite these efforts in identifying operational components or neuronal properties, the core computational principles of the cortex remain elusive (Krubitzer, 2009).

Here, we would like to suggest that the universal canonical computation performed by cortical circuits is the power-of-two-based, specific-to-general computational logic (Tsien, 2015). This logic is implemented vertically across cortical layers (Figure 2C). Using the moderately recurrent cortical architecture, the locally disordered but canonically nonrandom cortical patterns can readily execute this proposed logic. For example, the input-cortical layer, such as layer 4 (L4), hosts specific neural cliques. These layers’ pyramidal cells typically project upwards to layers 2 and 3 where some recurrent connections are made. This would produce two-event or three-event subgeneral cells (binary or ternary cliques). These subgeneral cells would then project to the deep layers, such as layers 5 and 6, for further combinatorial feature discovery and extraction to generate more broadly tuned subgeneral and general neural cliques. It should be noted that variations can occur; for example, some of the L5 neurons are directly driven by thalamic inputs. This type of direct input would enable specific features to still be maintained in the deep layers. Regardless of such variations in wiring details, the fundamental power-of-two-based computational logic remains invariant.

Not all cortices have the classic six layers, rather some cortices only use a three-layered cortex (e.g., the prefrontal cortex, anterior cingulate cortex, piriform cortex, or retrosplenial cortex in mice, and the entire cerebral cortex in reptiles; Fournier et al., 2015). In such three-layered cortices, classic layers 2 and 3 are merged in the upper layer (L2/3), whereas layers 5 and 6 form the deep layer (L5/6). In this scenario, specific cliques and binary subgeneral cliques should be enriched in L2/3, whereas the general cliques should be mostly in L5/6. Therefore, the power-of-two-based computational logic is still preserved in such three-layered architecture. In short, we propose that evolution relies on the multi-layered cortical architecture to execute the power-of-two-based mathematical principle. This explains why scaling up cognitive capacity and intelligence is achieved by dramatically expanding the cortical surface areas, rather than by varying the cortical thickness.

## To Test the *Theory of Connectivity* Experimentally

This theory can be tested initially by large-scale *in vivo* recordings, because structural connectivity is ultimately reflected by functional connectivity. One critical consideration is to identify the proper natural stimuli to which relevant cell assemblies in the higher cortex have been evolutionarily selected and developmentally programmed. Another consideration is to use multi-modality categorical stimuli (e.g., information *i* > 3), thereby allowing a more stringent testing of this power-of-two-of-two-based, specific-to-general computational logic in a given circuit. The proposed wiring and computational logic can be detected in the form of a brain activation “bar-code” (Figure 2D). This theory should be tested across many animal species—from fruit flies, zebra fish and songbirds, to rodents and primates (Gerber et al., 2004; Phillips et al., 2011; Beshel and Zhong, 2013; Shi et al., 2013; Tubon et al., 2013; Lin et al., 2014; Davis, 2015).

The theory further predicts that specific-to-general logic should be genetically programmed by evolution and during development and already takes its primitive shape, prior to learning, as the matured connectivity in the naïve unlearnt neural network. However, synaptic plasticity plays a crucial role in both neural development (Gao et al., 2014) and learning (Bliss and Collingridge, 1993; Frey and Frey, 2008). The critical question is how to distinguish the wiring logic set up by normal development vs. by learning and memory.

Because synaptic proteins are metabolically turned over within days or week(s), learning-induced synaptic connectivity will likely drift significantly over time (Shimizu et al., 2000; Wang et al., 2006). It has been shown that the NMDA receptor (NMDAR)-based synaptic reentry-reinforcement (SRR) is crucial for maintaining synaptic stability (Wittenberg and Tsien, 2002; Wittenberg et al., 2002). For example, inducible knockout of the NMDAR in the forebrain principal neurons for one-month (but not for one-week) caused a drift in the synaptic connectivity pattern, leading to the abolishment of remote fear memories (Cui et al., 2004). If the initial synaptic connectivity were random in the matured but unlearnt network, one should expect that deleting the NMDAR for a long period of time (e.g., 3 months) would eventually lead synaptic connectivity to drift all the way back to initial randomness. On the other hand, if one can still observe these non-random, specific-to-general neural cliques under such conditions, it would strongly suggest that this is a pre-configured logic and is independent of the NMDAR-dependent learning in adulthood.

## Installing the Personalized Cognitive Algorithms by Nature and Nurture

Changes in gene expression underlie brain development and aging (Jiang et al., 2001; Mody et al., 2001; Förster et al., 2006; Langston et al., 2010; Wills et al., 2010; Gao et al., 2014; He et al., 2015). This provides a unique opportunity to investigate not only how computational logic emerges during the postnatal period and is affected by aging, but also how environment alters or enhances cognition (Rampon et al., 2000a,b; van Praag et al., 2000; Feng et al., 2001; Tang et al., 2001).

The pre-configured connectivity, as proposed by the *Theory of Connectivity*, would conceivably constrain or give rise to different intelligence. It also predicts that variable ratios among the specific, subgeneral and general cliques among individual brains can afford distinct cognitive abilities or unique talents. The larger the number of neurons devoted to specific neural cliques, the greater the ability of remembering episodic details would be expected. In contrast, if more neurons were devoted to the subgeneral and general neural cliques, such individuals may possess a greater ability for abstraction, generalization or flexible behavior.

Genetic mutations can also alter the basic computational algorithms of neural circuits. By examining such interactions in various genetically modified mice—including NMDAR1 conditional knockout mice and the memory-enhanced NR2B transgenic mice (Tang et al., 1999; Cui et al., 2004; Wang et al., 2009; Yu et al., 2009; Jin and Costa, 2010; Zhang et al., 2013; Jacobs and Tsien, 2014), one can obtain crucial insight into the precise relationships between genes, neural circuits and cognition. In addition, Reeler mice can be a particularly interest model because of their inverted cortex (D’Arcangelo and Curran, 1998). One prediction might be that Reeler mice still operate under the power-of-two-based computational logic but with the inverted anatomical distributions of specific-to-general cliques. On the other hand, it is also possible that the cortex of Reeler mice may host the specific-to-general clique arrangement in a similar manner to wild-type mice. This would suggest that histological and molecular characteristics critical for defining the different cortical layers are not essential for executing the power-of-two-based, specific-to-general computational logic.

## Specific-To-General Cell Assembly for Representing Memory Engram

The study of memory engram has gained renewed interest. For example, using the C-fos promotor-based optogenetic approach, researchers reported a group of neurons in the DG or amygdala that were labeled during learning can be optogenetically reactivated during recall, leading to changes in freezing (see reviews by Josselyn et al., 2015; Tonegawa et al., 2015). Intriguingly, the same protocols and manipulation in the CA1 failed to produce a similar outcome produced by the DG (Xu Liu, personal communication), thereby raising the possibility that the artificial zapping of the amygdala loop by light may not truly alter memory engram *per se*. In general, methods using immediate-early-gene promoter to label memory engram are interesting, but it lacked the ability to distinguish categorical features and to obtain temporal dynamics essential for revealing the fundamental principles and encoding properties of memory engram (see Eichenbaum, 2016).

Memory is traditionally divided into episodic memory and semantic memory. Episodic memory refers to the memory of a specific event in a given time and context (Tulving, 1972), whereas semantic memory represents the memory of the conceptual knowledge of facts that are no longer ascribable to any particular occasion in life (Tulving, 1972; Cohen and Eichenbaum, 1993; Squire and Zola, 1998; Lin et al., 2007). This classical definition has led to intense search for the distinct network-mechanisms underlying the formation of episodic and semantic memory (Düzel et al., 1999; Maguire et al., 2005; Burianova and Grady, 2007; Ryan et al., 2008). Yet it remains unclear as to how semantic memory emerges from daily experiences, and what the relationship is between these two types of memories at the cell-assembly level.

The initial clue is provided by the revelation of specific-to-general neural clique assembly in the CA1 hippocampus (Lin et al., 2005, 2006; Tsien, 2007), which suggested that episodic memory and semantic memory are simultaneously generated within the same cell assembly. In other words, the computational logic used by memory engram follows the same power-of-two-based mathematical principle: the specific cliques extract specific features from sensory information to encode episodic traces of the memory engram, whereas the subgeneral and general cliques generate the relational knowledge and concepts of the memory engram (Tsien et al., 2013). Such simultaneous extractions of both episodic and semantic memory components by the same FCM offer a perfect solution to build the categorical and hierarchical organization of memory and knowledge in the brain. Encouragingly, large-scale neural recording experiments have begun to uncover critical insights and temporal patterns of real-time memory traces and fear memory engram in the normal brain as well as in the absence of synaptic plasticity (Chen et al., 2009; Oşan et al., 2011; Zhang et al., 2013).

## General-To-Specific Combinatorial Logic for Motor Output Circuits

Gradually extracting perceptual information by sensory and memory circuits is what leads to generalized knowledge and concepts. In contrast, the motor output circuits may use the same specific-to-general computational logic—but in reverse. That is, cell assemblies in higher motor circuits (such as the motor-planning cortex) will be more general and abstract to begin with, then percolate to primary cortices and lower circuits, which become successively more specific. Motor-control circuits are crucial for a broad range of functions, spanning from movement planning and execution to imagined movement or motor cognition (Bizzi et al., 1995; Nicolelis et al., 1997; Moran and Schwartz, 1999; Fogassi et al., 2005; Georgopoulos and Carpenter, 2015; Stetson and Andersen, 2015). Overall, general-to-specific cliques in the FCM at the top of the hierarchy encode more abstract intent or decision-making for general motor execution, whereas the cliques in the lower FCMs encode less abstract motor command, with FCMs in spinal circuits commanding general-to-specific individual muscle activation. One can test these predictions by large-scale recording in the motor cortex, striatum, and spinal cord.

## FCM as the Key Biomarker for Assessing Neurological and Psychiatric Disorders

The proposed theory should open a new avenue into examining the detrimental effects of brain disease and aging at the cell-assembly level. Characterizations of cell-assembly patterns in various diseased models—from Alzheimer’s to depression (D’Arcangelo and Curran, 1998; Hayashi et al., 2008; Feng et al., 2001)—can potentially lead to deeper insights into why gene mutations alter cognitive functions. In addition, examining the effects of various drugs on the proposed cell-assembly logic may provide new insights about their central mechanisms (Caine et al., 2007; Wang et al., 2010; Kong and Xu, 2011; Cao et al., 2015; Fuccillo et al., 2016), perhaps leading to further improvement for drug efficacy and safety (Slutsky et al., 2010; Liu et al., 2015).

In summary, the *Theory of Connectivity* postulates the *power-of-two*-based, *specific-to-general* wiring and computational logic for the organization of pre-configured cell assemblies in the brain. This prediction is radically different from local random connectivity currently assumed for cell assemblies in matured, but unlearnt, circuits. This theory also provides a new framework to investigate how learning and development interact to produce generative cognition and flexible behavior.

## Author Contributions

JZT developed the idea and worked with ML and JL, as well as co-wrote the manuscript.

## Funding

We would like to express our gratitude for the unique opportunity of working at the Brain Decoding Project Consortium organized by Banna Biomedical Research Institute of Yunnan Academy of Science and Technology and supported by Yunnan Science Commission (2014DG002). We also thank Georgia Research Alliance for its support of the Brain Decoding Project and National Institute of Health (R01NS079774) to JT.

## Conflict of Interest Statement

The authors declare that the research was conducted in the absence of any commercial or financial relationships that could be construed as a potential conflict of interest.
